# Probiotic Yeasts and How to Find Them—Polish Wines of Spontaneous Fermentation as Source for Potentially Probiotic Yeasts

**DOI:** 10.3390/foods12183392

**Published:** 2023-09-11

**Authors:** Adam Staniszewski, Monika Kordowska-Wiater

**Affiliations:** Department of Biotechnology, Microbiology and Human Nutrition, University of Life Sciences in Lublin, Skromna 8, 20-704 Lublin, Poland; adam.staniszewski@up.lublin.pl

**Keywords:** enology, Polish wines, probiotics, *Saccharomyces cerevisiae* var. *boulardii*, probiotic yeasts, non-Saccharomyces yeast, viniculture

## Abstract

One approach towards maintaining healthy microbiota in the human gastrointestinal tract is through the consumption of probiotics. Until now, the majority of probiotic research has focused on probiotic bacteria, but over the last few years more and more studies have demonstrated the probiotic properties of yeast, and also of species besides the well-studied *Saccharomyces cerevisiae* var. *boulardii*. Probiotic strains have to present the ability to survive in harsh conditions of the host body, like the digestive tract. Must fermentation might be an example of a similar harsh environment. In the presented study, we examined the probiotic potential of 44 yeast strains isolated from Polish wines. The tested isolates belonged to six species: *Hanseniaspora uvarum, Pichia kluyveri, Metschnikowia pulcherrima, Metschnikowia ziziphicola, Saccharomyces cerevisiae* and *Starmerella bacillaris*. The tested strains were subjected to an assessment of probiotic properties, their safety and their other properties, such as enzymatic activity or antioxidant properties, in order to assess their potential usefulness as probiotic yeast candidates. Within the most promising strains were representatives of three species: *H. uvarum*, *M. pulcherrima* and *S. cerevisiae*. *H. uvarum* strains 15 and 16, as well as *S. cerevisiae* strain 37, showed, among other features, survivability in gastrointestinal tract conditions exceeding 100%, high hydrophobicity and autoaggregation, had no hemolytic activity and did not produce biogenic amines. The obtained results show that Polish wines might be a source of potential probiotic yeast candidates with perspectives for further research.

## 1. Introduction

The human microbiota significantly influence the well-being of the human host, and may participate in the development of a wide variety of diseases. One of the approaches towards maintaining healthy microbiota is through probiotic consumption [1,2]. The World Health Organization defines probiotics as live microorganisms that, when administered in sufficient amounts, confer a health benefit. While the majority of probiotic research has focused on bacterial strains (mainly *Lactobacillus* (according to previous nomenclature) and *Bifidobacterium*), the exploration of probiotic yeast is just gaining momentum due to its potential in terms of unique features and potential therapeutic applications [3,4,5]. Most probiotic yeast research focuses on *Saccharomyces cerevisiae* var. *boulardii*, the only species of yeast that has been tested well and is widely used as a probiotic [6,7,8,9]. However, more and more research is focused on the search for new strains with probiotic properties, not only *Saccharomyces cerevisiae* var. *boulardii* [4,10,11,12]. Examples of non-Saccharomyces species with probiotic properties may be *Debaryomyces hansenii* and *Kluyveromyces marxianus* [13,14,15].

Fermented foods and beverages have been a source of probiotic microorganisms in the human diet since the dawn of time. The coexistence of yeasts and lactic acid bacteria often occurs in a synergistic manner, where they mutually enhance their growth and survival [16,17,18]. Examples of fermented products that can be sources for the isolation of probiotic strains are cheese, dough, dairy products, fermented fruits and vegetables, and traditional food and beverages [19,20,21]. Among such products, wines deserve attention as a source of probiotic microorganisms due to the richness of their microbiota [22,23,24,25,26,27,28]. Examples of such potentially probiotic isolates include bacteria like *Lactobacillus fermentum*, *Lactobacillus rhamnosus*, *Pediococcus pentosaceus* [23,29] and both *Saccharomyces* and non-Saccharomyces yeasts like *Candida* spp., *Hanseniaspora* spp., *Pichia* spp. and *Torulaspora delbrueckii* [27,28,30]. Over recent years, with the warming of Poland’s climate, there has been an increased interest in viticulture, resulting in the growth of vineyards by over 550 in the last decade [25,31]. At the time of publication preparation, only two publications characterizing native yeast strains of Polish wines and one metagenomic study were available, but none of them were dedicated to exploring the probiotic potential of the tested strains [22,25,32]. To the best of our knowledge, this is the first study focusing on the aspect of probiotic potential of yeast isolates derived from Polish wines and may provide some new perspectives on wine-derived yeasts besides the typical look inside their influence on wine’s enological properties, such as aroma, acidity, texture, etc.

The main aim of the study was the isolation of probiotic yeast candidate strains from spontaneously fermented Polish wines and the in vitro testing of their potentially probiotic and safety properties.

## 2. Materials and Methods

### 2.1. Yeast Strains

Forty-four yeast strains were obtained from the culture collection of the Department of Biotechnology, Microbiology and Human Nutrition, Faculty of Food Science and Biotechnology, University of Life Sciences in Lublin. The strains originated from spontaneously fermented wines produced from grapes of the Regent variety sourced from three Polish vineyards, “Dom Bliskowice” (DB), “Małe Dobre” (MD) and “Winnica Janowiec” (WJ), located in the Małopolska Vistula Gorge region in 2019. Fresh cultures for the following experiments were grown for 48 h at 28 °C in YPD (yeast extract peptone dextrose) broth (BTL, Łódź, Poland). A commercial probiotic yeast, *Saccharomyces cerevisiae* var. *boulardii* CNCM I-745 (Enterol, Biocodex, France), was used as a positive control. To ensure consistency in the measurements, the initial optical density at 600 nm (OD_600_) of each culture was determined. Subsequently, the results were standardized to an OD_600_ value of 1.0 for each experiment, unless stated otherwise. This normalization allowed for accurate and comparable data analysis across different cultures. The strains were preserved in freezing conditions (−20 °C) with glycerol (20%). The code names for individual strains with vineyard origin are listed in Table 1.

#### Yeast Identification

To verify the identification of the strains obtained from the collection, they were subjected to species identification based on ITS1-5.8S rDNA-ITS2 regions. Strains’ identification was obtained via 5.8S-ITS gene sequencing. DNA isolation was performed according to the procedure of Genomic Mini AX Yeast (A&A Biotechnology, Gdańsk, Poland). Amplification of the region was performed with the primers ITS1 (5′TCCGTAGGTGAACCTGCGG-3′) and ITS4 (5′-TCCTCCGCTTATTGATATGC-3′) using PCR Mix Plus Green (A&A Biotechnology, Gdańsk, Poland) with both primers concentration 0.5 µM and 1 µL of fungal DNA template [33]. Amplification conditions: cycle of initial denaturation at 95 °C for 5 min, followed by 34 cycles: denaturation at 95 °C for 1 min, annealing at 56 °C for 1 min, extension at 72 °C for 2 min with final extension step at 72 °C for 10 min. Sequencing of the amplicons was performed using Genomed (Warsaw, Poland). Contigs were assembled with DNA Sequence Assembler v5 (Heracle BioSoft, www.DnaBaser.com) (accessed on 10 January 2023). Obtained contigs were compared to the known sequences of the ITS region in the NCBI GenBank database via alignment with the BLAST algorithm and evolutionary analyses were conducted in MEGA11. The phylogenetic tree was created based on the ITS region sequences using the maximum likelihood method with the best-fit Kimura 2-parameter model with a discrete gamma distribution (+G), with 1000 bootstrap replication [34].

### 2.2. Evaluation of Potentially Probiotic Traits

#### 2.2.1. Survival and Growth at 37 °C

For checking the ability to grow at human body temperature, 10 μL of fresh yeast culture was inoculated into 1 mL of YPD (Yeast Peptone Dextrose) broth and was incubated at 37 °C for 48 h. Survival under these conditions was assessed via growth or no growth after 48 h. If the strain did not show the ability to grow in the above-mentioned conditions, it was removed from further analysis.

#### 2.2.2. Survival and Growth under Gastrointestinal Tract Conditions

All strains undergo in vitro digestion according to Fernandez-Pacheco et al., 2018, with modification [12]. One milliliter of fresh culture was centrifuged (5000 rpm, 10 min), biomass was washed with YPD broth in PBS (phosphate-buffered saline) at pH = 2 supplemented with 3 mg/mL pepsin, centrifuged under the same conditions and after washing, inoculated into 1 mL of gastric solution containing YPD supplemented with 3 mg/mL pepsin in phosphate-buffered saline at pH = 2 to simulate gastric conditions. The mixture was then incubated at 37 °C for 3 h. Following this, the gastric solution was centrifuged (5000 rpm, 10 min) and the biomass was washed with an intestinal solution composed of YPD broth containing 1 mg/mL pancreatin and 0.5% bile salts, adjusted to pH = 8 and was transferred to the same intestinal solution and incubated at 37 °C for 22 h. To assess survival and growth of cells under gastrointestinal conditions, the spread plate technique on YPD agar was used after serial dilution preparations [35,36]. 

Survival rate and growth was calculated as % by the formula:

survival rate [%] = concentration of living cells after in vitro digestion/ concentration of living cells before in vitro digestion × 100%

#### 2.2.3. Hydrophobicity of Cell Surface

Cell surface hydrophobicity was tested according to Amorim et al., 2018 [37]. One milliliter of fresh culture was centrifuged (5000 rpm, 10 min), biomass was washed twice and was resuspended in 5 mL of PBS (phosphate-buffered saline) at pH = 7. Three milliliters were blended with 1 mL of xylene. The mixture was vigorously shaken for 2 min and then allowed to settle undisturbed at 37 °C for 30 min, facilitating the complete separation of the phases. Subsequently, the aqueous phase was carefully removed, and the absorbance at 600 nm was measured spectrophotometrically. The decrease in the absorbance was taken as the measure of cell surface hydrophobicity calculated using the formula bellow:

Hydrophobicity [%] = [1 − ((OD_initial_ − OD_final_)/OD_initial_)] × 100%

where OD_initial_ and OD_final_ are the absorbance (at 600 nm) before and after extraction with xylene [37,38,39].

#### 2.2.4. Autoaggregation Assay

Autoaggregation assay was carried out according to Amorim et al., 2018, and Gil-Rodriguez et al., 2015 [37,40].

The yeasts were grown in YPD broth at 37 °C for 24 h. After that, they were centrifuged (5000 rpm, 10 min) and washed twice with PBS at pH = 7. The biomass was then resuspended in PBS. To assess autoaggregation, 3 mL of the cell suspension was vortexed for 10 s. Autoaggregation was determined spectrophotometrically after 2, 4 and 24 h of incubation at 37 °C via absorbance (A) (OD_600_) and it was expressed as:

Autoaggregation [%] = [1 − (A_t_/A_0_] × 100%

where A_t_ is the absorbance at 2, 4 or 24 h and A_0_ is at zero time [37].

#### 2.2.5. Antioxidant Activity 

Antioxidant activity assay was carried out according to Gil-Rodriguez et al., 2015 [40]. One milliliter of yeast culture in YPD broth was centrifuged (5000 rpm, 10 min), washed twice with a sterile solution 0.9% NaCl and the pellet was resuspended in 1 mL of 0.9% NaCl. Next, 800 μL of the cell suspension was transferred to a new tube, to which 1 mL of a DPPH (2,2-diphenyl-1-picrylhydrazyl) solution (0.2 mM in methanol) was added. The mixture was vortexed and incubated in darkness at room temperature for 30 min. Following incubation, the reaction tubes were centrifuged (12,000 rpm, 5 min) and 300 μL of the resulting supernatant was transferred to 96-well plates for measurement of the absorbance at 517 nm (A_517_). The percentage of reduction in DPPH was then calculated using the following formula [40]:

Percentage of reduction of DPPH [%] = [(A_517 control_ − A_517 sample_)/A_517 control_] × 100%

where A_517 control_ represents the absorbance of the control (DPPH solution without yeast) and A_517 sample_ represents the absorbance of the sample (yeast culture treated with DPPH solution).

#### 2.2.6. Antimicrobial Activity

The well-diffusion method was employed to evaluate the antimicrobial activity of the yeast strains against various bacterial species [41,42], including *Escherichia coli*, *Salmonella enterica*, *Staphylococcus aureus*, *Bacillus cereus*, *Listeria monocytogenes* and *Enterococcus faecalis*. The bacterial strains were cultivated in BHI (brain heart infusion) broth at 37 °C overnight. Then, the bacterial cultures were individually inoculated with 1mL of bacterial suspension onto BHI agar plates, spread and left to allow for the liquid absorption. Afterward, three wells (5 mm diameter each) were cut in the center of every plate at equal intervals between each other and inoculated with fresh yeast culture. The plates were incubated for 24 h at 37 °C, to evaluate bacterial growth inhibition.

### 2.3. Traits Related to Safety and Virulence

#### 2.3.1. Hemolytic Activity

To test hemolytic activity, yeasts were streaked onto blood agar plates (Columbia agar supplemented with 5% defibrinized sheep’s blood) (Biomaxima, Lublin, Poland). The plates were then incubated at 37 °C for 72 h. Following incubation, the plates were observed for any visible signs of hemolysis. As a positive control, *Staphylococcus aureus*, known to exhibit hemolysis, was included in the experiment.

#### 2.3.2. Biogenic Amine Production

To evaluate strains’ ability to produce biogenic amines, the method suggested by Aslankoohi et al., 2016 was used [43]. Yeasts were streaked onto YPD agar plates supplemented with 0.006% bromocresol purple and an amino acid mix (tyrosine, histidine, phenylalanine, leucine, tryptophan, arginine and lysine in equal ratios) with a total mass concentration of 1%. For the detection of biogenic amines (BAs), the plates were incubated at 30 °C for 7 days. Throughout the incubation period, the growth of strains and any changes in the color of the medium were monitored on a daily basis. This monitoring aimed to identify the presence of biogenic amines, which could be indicated by specific changes in the appearance of the medium. In yeast strains that produce biogenic amines, the process of amino acid decarboxylation resulted in the immediate appearance of a purple halo surrounding the growth area. On the other hand, in the strains that did not produce biogenic amines, the growth area exhibited a yellow halo surrounding it, which was attributed to glucose fermentation. During the growth of the strain, pH reduction occurred, leading to the medium gradually turning purple [43].

### 2.4. Enzymatic Activity

We determined the enzymatic activities of the strains using the API ZYM system (bio-Merieux, Craponne, France) according to the manufacturer’s recommendations. Yeast cell suspensions were transferred into the wells of the API ZYM strips and incubated at 37 °C for 4 h. After the incubation period, one drop each of the reagents ZYM A and ZYM B was added to each well. Color changes observed in the wells indicating positive enzymatic reactions were noted and used for evaluation of the results on the basis of the API ZYM color chart [44].

### 2.5. Statistical Analysis

The results were analyzed with Statistica version 13.3 (2017) for Windows (StatSoft Inc., Tusla, OK, USA). The one-way ANOVA followed by Tukey HSD post hoc tests (*p* < 0.05) was used to compare the results for: survivability, hydrophobicity and antioxidant activity for each strain, and mixed two-way ANOVA for level of autoaggregation for testing strains after 2, 4 and 24 h. 

## 3. Results

### 3.1. Yeast Identification

**Figure 1:**
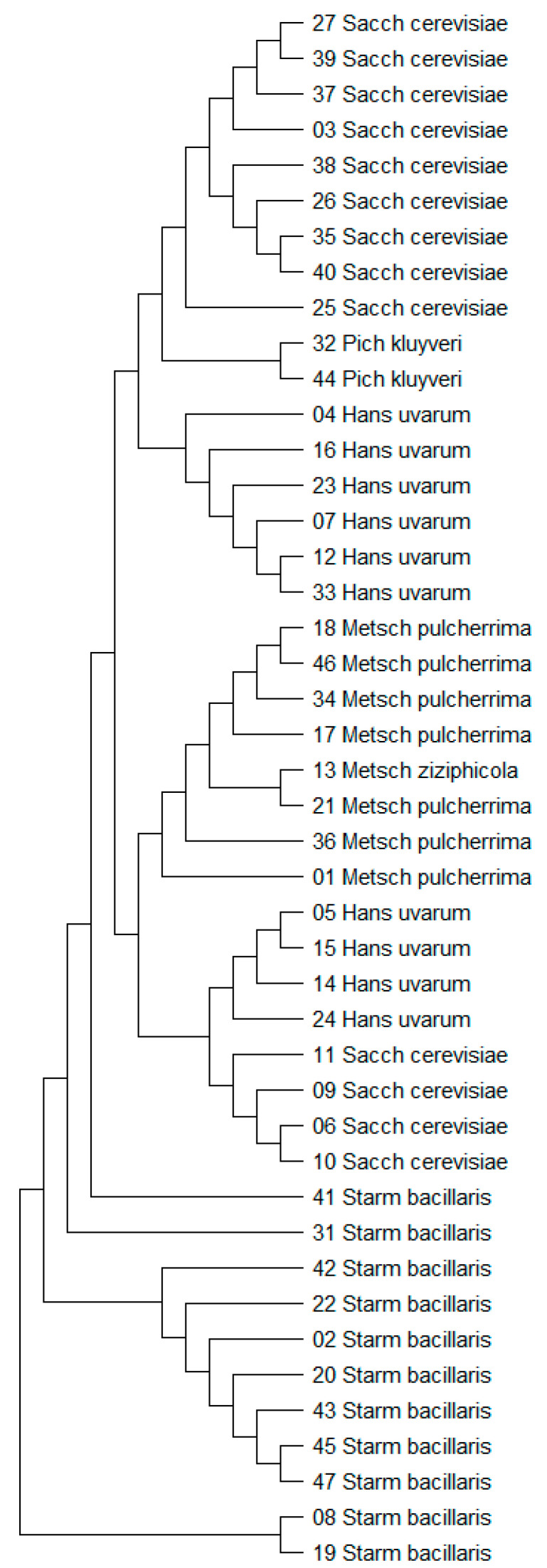
Maximum likelihood tree based on ITS sequences of the studied isolates.

All strains were identified to the species level (Table 1). All forty-four identified strains belong to five yeast genera: *Saccharomyces* (29.54% of strains)*, Starmerella* (25% of strains), *Hanseniaspora* (22.72% of strains), *Metschnikowia* (18.2% of strains) and *Pichia* (4.5% of strains). The results of the phylogenetic analysis are presented in Figure 1.

### 3.2. Evaluation of Potentially Probiotic Traits

#### 3.2.1. Survival and Growth at 37 °C

**Figure 2:**
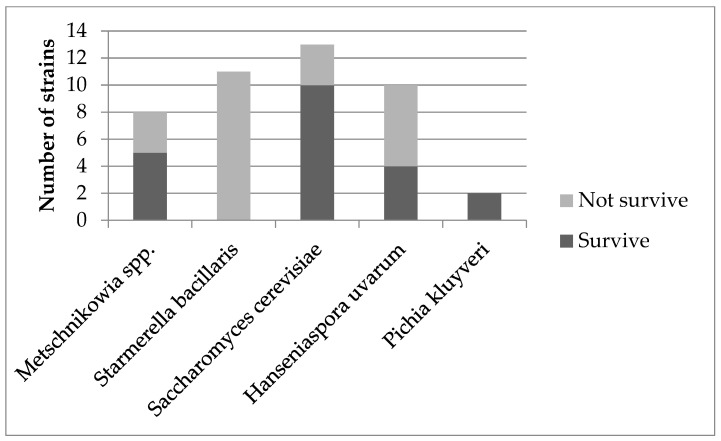
Ability to survive at 37 °C.

Out of forty-four tested strains only twenty-one isolates showed an ability to survive and grow at 37 °C. Within tested strains, the highest percentage of survived strains was shown by *Pichia kluyveri* (100% of strains survived), followed by *Saccharomyces cerevisiae* (76.9% of strains survived), *Metschnikowia* spp. (62.5% of strains survived) and *Hanseniaspora uvarum* (40% of strains survived). None of the tested *Starmerella bacillaris* isolates survived. Detailed data are presented in Figure 2. All strains that did not demonstrate the ability to grow at 37 °C were excluded from further studies. 

#### 3.2.2. Survival and Growth under Gastrointestinal Tract Conditions

**Figure 3:**
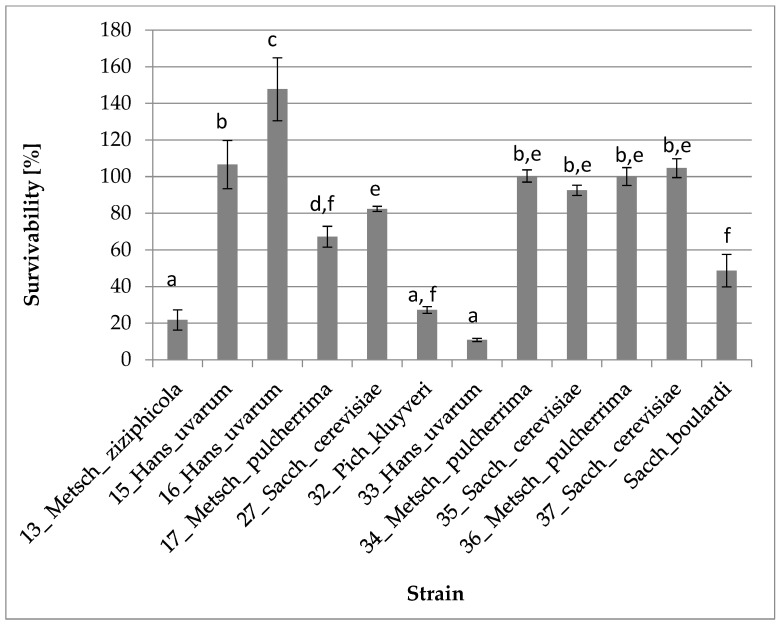
Survival and growth of yeasts under gastrointestinal tract conditions (one-way ANOVA: strain: F_(11,24)_ = 89,533, *p* = 0.00000; se ± 0.043683). The values designated by the different letters are statistically significantly different.

As the next step, the remaining strains were subjected to in vitro digestion. Out of twenty-one strains subjected to the procedure, eleven strains showed the ability to survive simulated gastrointestinal conditions. Eight of the eleven strains showed better survivability than the positive control (*Saccharomyces cerevisiae* var. *boulardii* CNCM I-745 with survivability at 48.7%). Five strains demonstrated survivability above 100%: 16_Hans_uvarum (147.8%), 15_Hans_uvarum (106.6%), 37_Sacch_cerevisiae (104.6%), 34_Metsch_pulcherrima (100.4%) and 36_Metsch_pulcherrima (100.1%). This may suggest that the conditions in the gastrointestinal tract for the five mentioned strains could be prevailing for their growth compared to standard culture conditions. Detailed data are presented in Figure 3. All strains that did not demonstrate the ability to survive gastrointestinal tract conditions were excluded from further studies and are not included in Figure 3.

#### 3.2.3. Hydrophobicity of Cell Surface

**Figure 4:**
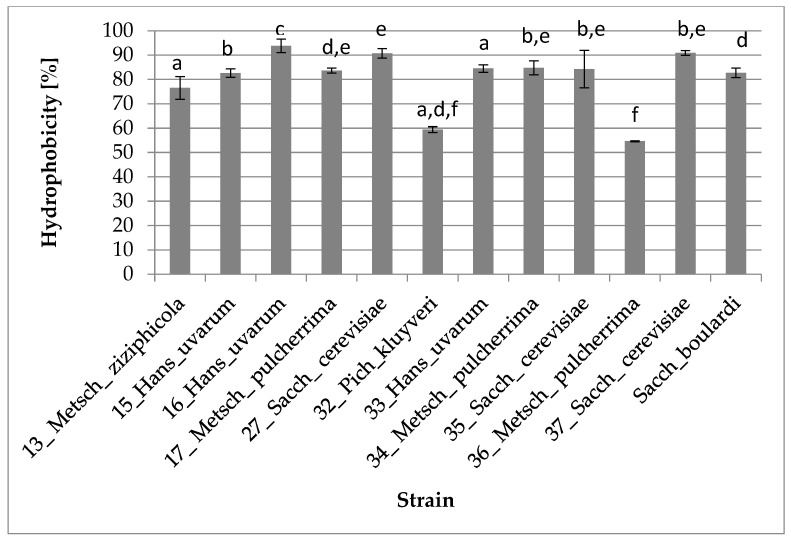
Hydrophobicity of cell surface of yeasts (one-way ANOVA: strain: F_(11,24)_ = 45,775, *p* = 0.00000; se ± 0.017750). The values designated by the different letters are statistically significantly different.

The ability to adhere to intestinal epithelial cells is a crucial requirement for the colonization of potentially probiotic strains in the gastrointestinal tract, as it helps them avoid immediate elimination through peristalsis and gives a competitive advantage within the gastrointestinal tract. Many authors suggest that strains with high hydrophobicity exhibit high adherence to intestinal cell lines [45,46]. All, except three strains: 36_Metsch_pulcherrima (54.63%), 32_Pich_kluyveri (59.42%) and 13_Metsch_ziziphicola (76.53%), showed hydrophobicity above 80% which may suggest their ability to quickly adhere to the mucosa. Detailed data are presented in Figure 4.

#### 3.2.4. Autoaggregation Assay

**Figure 5:**
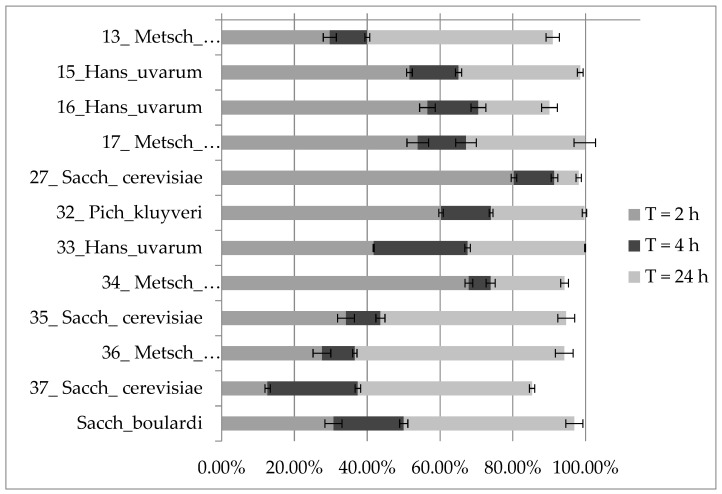
The results of autoaggregation assay for each strain after 2, 4 and 24 h (two-way ANOVA: strain: F_(11,72)_ = 925.80, *p* = 0.00000; se ± 0.431909; time: F_(2,72)_ = 13,991, *p* = 0.00000; se ± 0.215954; strain × time: F_(22,72)_ = 174.89, *p* = 0.00000; se ± 0.748088).

Pizzolitto et al., 2013, and Amorim et al., 2018, suggest that autoaggregation above 80% should be considered as high [37,47]. Besides 37_Sacch_cerevisiae, all strains presented autoaggregation above 25% after 2 h with the highest autoaggregation rate after 2 h for strains: 27_Sacch_cerevisiae (80.31%), 34_Metsch_pulcherrima (67.90%) and 32_Pich_kluyveri (60.28%). For all strains, autoaggregation rates increased with time reaching above 90% after 24 h, excluding 37_Sacch_cerevisiae, which obtained autoaggregation rates at 12.62%, 37.42% and 85.30% after 2, 4 and 24 h. Detailed data are presented in Figure 5. Result of Tukey HSD post hoc test for Figure 5 is included in the Supplementary Materials (Spreadsheet S1: Figure_5_Tukey_results).

#### 3.2.5. Antioxidant Activity 

**Figure 6:**
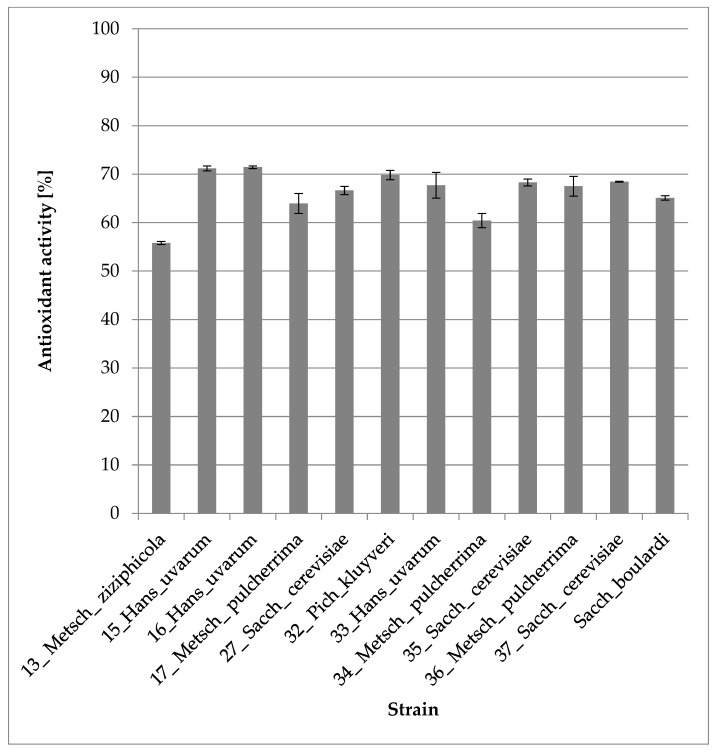
Antioxidant activity for tested strains (one-way ANOVA: strain: F_(11,24)_ = 36,078, *p* = 0.00000; se ± 0.007562).

The DPPH assay showed that all tested strains presented high antioxidant activity, but only five strains: 13_Metsch_ziziphicola (55.79%), 15_Hans_uvarum (71.20%), 16_Hans_uvarum (71.44%), 32_Pich_kluyveri (69.83%) and 34_Metsch_pulcherrima (60.42%) showed a significant difference in antioxidant activity toward the control sample of Sacch_boulardi (65.10%). Detailed data are presented in Figure 6. Result of Tukey HSD post hoc test for Figure 6 is included in the Supplementary Materials (Spreadsheet S1: Figure_6_Tukey_results).

#### 3.2.6. Antimicrobial Activity

Only strain 15_Hans_uvarum showed weak antimicrobial activity against *Staphylococcus aureus*; other strains did not inhibit the growth of pathogenic bacteria. 

### 3.3. Traits Related to Safety and Virulence

#### 3.3.1. Hemolytic Activity

None of strains tested in the experiment presented hemolytic activity.

#### 3.3.2. Biogenic Amine Production

From all tested strains, three strains: 27_Sacch_cerevisiae, 32_Pich_kluyveri and 34_Metsch_pulcherrima produced a purple halo around the growth area, which indicate the presence of biogenic amines [43]. 

### 3.4. Enzymatic Activity

The API ZYM assay showed various activity profiles within the tested strains. All strains presented activity of alkaline phosphatase, esterase (C4), esterase lipase (C8) and acid phosphatase, but none of the strains presented activity of trypsin, α-galactosidase, β-glucuronidase nor α-fucosidase. The differences in enzymatic activity between tested strains are presented in Table 2. 

## 4. Discussion

At the time of preparation for publication, to the best of our knowledge, nobody has published studies focused on the aspect of probiotic potential of yeasts isolated from Polish wines.

The forty-four strains examined belonged to six species: *Hanseniaspora uvarum*, *Pichia kluyveri*, *Metschnikowia pulcherrima*, *Metschnikowia ziziphicola*, *Saccharomyces cerevisiae*, and *Starmerella bacillaris*. Despite limited research on yeast biodiversity in Polish wines, our findings mostly align with the lists of species described by Drozdz et al. and Cioch-Skoneczny et al. [22,32], but include one species not mentioned by the authors—*Starmerella bacillaris*. The species, *S. bacillaris*, known also as *Candida zemplinina*, is frequently isolated from grapes and wines, and may affect the chemical composition of the musts and wines by its ability to produce various metabolites which influence their enological properties [48,49,50]. All species identified in the study commonly occur and are isolated from grape, must and wine environments worldwide [51,52,53]. 

Despite challenging environmental conditions for microorganisms during the wine fermentation process, only 11 out of the 44 strains subjected to the initial experiments demonstrated potential for further research. Two of the most discriminative tests for the experiments were the ability to survive at 37 °C and the ability to survive and grow under gastrointestinal tract (GIT) conditions, eliminating 52.3% and 47.6% of the strains at each of those steps. Among the isolates able to survive the two initial steps of the experiment were the strains belonging to *H. uvarum, P. kluyveri, M. pulcherrima, M. ziziphicola* and *S. cerevisiae*. Besides their enological potential, none of *S. bacillaris* strains tested in the study were able to survive and grow at 37 °C, which stands in opposition to the results obtained by Shen et al., who described *S. bacillaris* CC-PT4 as showing properties as a probiotic candidate [54]. Current data about potentially probiotic strains belonging to *S. bacillaris* are limited to the data of Shen et al. and do not include exact information about the source of isolation of each strain and the information is mainly limited to the list of fruits bought from greengrocers in China. Possibly the reason for differences in the tolerance for the host temperature and the ability to survive under GIT conditions between *S. bacillaris* strains from this study and Shen et al.’s study might be differences in climate conditions at the place of isolate’s origin, but such a hypothesis cannot be verified without more detailed data.

Gastrointestinal tract conditions play an important role in human health protection. serving as a barrier between the external environment and the body and helping to prevent the entry of harmful substances and pathogens. Such conditions also play important role in survival of potential probiotics [40]. In our study, eight of the eleven strains presented better survival rate than *Saccharomyces cerevisiae* var. *boulardii* CNCM I-745 (positive control), with survivability at 48.7%. Two strains for both *Hanseniaspora uvarum* and *Metschnikowia pulcherrima*, and one strain of *Saccharomyces cerevisiae* exceeded 100% survival rate, which may suggest that GIT conditions are more favorable for them than typical culture conditions. Similar results for wine-derived strains were published by Vergara Alvarez et al. [27]. Such properties may result from the adaptation of the strains to the conditions of wine fermentation, where low pH is common [55,56].

Hydrophobicity and autoaggregation assays allow for the estimation of microorganisms’ behavior in GIT [12]. Yeasts with high hydrophobicity exhibit high adherence to intestinal cell lines [38,39]. All strains, with exception of 13_Metsch_ziziphicola, 32_Pich_kluyveri and 36_Metsch_pulcherrima, showed similar or higher hydrophobicity to the control strain, with the highest hydrophobicity in 16_Hans_uvarum (93.83%), 27_Sacch_cerevisiae (90.70%) and 37_Sacch_cerevisiae (90.87%). 

Pizzolitto et al. suggest that an autoaggregation rate at 80% or higher should be considered as high [47]. For all examined strains, autoaggregation rates increased with time exceeding above 80% after 24 h and were higher than values obtained for strains derived from various food environments tested by Fernandez-Pacheco [12]. Similar results for wine-derived strains were presented by Vergara Alvarez et al. [27].

According to Gil-Rodriguez et al., 2015, the antioxidant activity of yeasts can be a result of their cell walls and other cellular compounds and sometimes even higher than in lactic acid bacteria [40]. In the study, antioxidant activity levels of tested isolates were close to those of the control, *S*. *cerevisiae* var. *boulardii.*

Despite some yeast strains exhibiting antimicrobial properties (i.e., *M. pulcherrima* known for its ability to produce antimicrobial pulcherrimin), none of the tested strains showed such properties against tested pathogenic bacteria, which was consistent with the results of other researchers [30,57,58]. 

As a safety aspect, no hemolytic activity was present in our wine-derived strains. That is a result comparable to those obtained by Corbu et al., 2023, and Fernández-Pacheco et al., 2021 [59,60]. However, the strains 27_Sacch_cerevisiae, 32_Pich_kluyveri and 34_Metsch_pulcherrima showed the ability to produce biogenic amines (BAs) that excludes them as candidates for potentially probiotic strains [43], despite their promising properties in previous assays, due to potential risks for consumers [61,62]. Both Caruso et al., 2001, and Delgado-Ospina et al., 2021, showed that strains belonging to *M. pulcherrima*, *S. cerevisiae* and *P. kluyveri* might produce BAs [63,64]. Comparing the number of the strains producing BAs to the total number of strains in their and our study (where only three of all strains produced BAs) the result can be surprising, especially in the context of places of isolation—wine, in which the presence of biogenic amines formed during the fermentation process is not unusual and influences the sensory properties of wine. Our findings are closer to results obtained by Landete et al., 2007, where none of the examined wine-derived yeast strains produced BAs [65].

All tested strains presented a wide range of enzymatic activity including enzymes that break down proteins and sugars like esterases, lipase, arylamidases and α-glucosidase, which may confer a potential improvement on food digestion. Moreover, none of the strains presented any activity of trypsin (the enzyme that can be related with pathogenicity of some microorganisms in human) or β-Glucuronidase related to transformation of pre-carcinogens into carcinogens and stimulation of colon cancer [66,67]. Activity of enzyme aminopeptidases like arylamidases and esterases may influence product properties because of their biotransformation ability [21,67,68,69]. Four strains also presented activity of N-acetyl-β-glucosaminidase, an enzyme with the primary functions of targeting and hydrolyzing oligosaccharides containing chitin [70] that may possibly arise in the biocontrol and biotransformation of some fungal- and invertebrate-based foods. 

## 5. Conclusions

Our data show that Polish wines may be a source of yeast isolates with probiotic potential. Environmental conditions that occur during the must fermentation can help to preselect yeast for some desirable probiotic traits like low pH tolerance. Although isolates obtained during the study were sensitive for high temperature, 52.3% of strains were not able to grow and survive at 37 °C. One of the potential hypotheses of such a result may be due to the climate in Poland, which, despite global warming, does not force the local yeast populations to evolve towards higher temperature tolerance, but further studies will be needed. 

The strains with the highest resistance to the conditions in the gastrointestinal tract belong to the species: *Hanseniaspora uvarum* (15_Hans_uvarum and 16_Hans_uvarum)*, Metschnikowia pulcherrima* (34_Metsch_pulcherrima and 36_Metsch_pulcherrima) and *Saccharomyces cerevisiae* (37_Sacch_cerevisiae). These strains exceeded the 100% survival rate at GIT conditions and at least doubled the survival rate showed by commercial probiotic yeast *Saccharomyces cerevisiae* var. *boulardii* CNCM I-745, which suggests their high adaptation to such harsh conditions and may allow a reduction in dosage in the case of probiotic administration.

Besides their ability to survive in GIT conditions, four of the five mentioned isolates showed high hydrophobicity and autoaggregation, which are highly desirable traits in probiotic strains, with the exception of 36_Metsch_pulcherrima with the lowest hydrophobicity rate among all tested strains. Antioxidant activities of tested isolates were close to the control *S*. *cerevisiae* var. *boulardii.* Unfortunately, the strains did not show the ability to inhibit the growth of common foodborne pathogens.

The selected strains showed valuable enzymatic activities necessary in food digestion and did not have activities of undesirable enzymes such as trypsin and β-glucuronidase.

As a safety aspect, none of the strains presented hemolytic activity, but 34_Metsch_pulcherrima produced biogenic amines; thus, regardless of its other promising characteristics, it cannot be considered as a probiotic yeast candidate. 

Here, we can suggest some improvements in the experimental design in future—in case of screening high number of isolates, we suggest screening the tolerance for both temperature and GIT condition at first and moving forward to testing the traits related to the virulence and safety aspects, which will allow a reduction in costs and workload, in the case of experiments where high number of isolates may present such undesirable traits.

In our opinion, Polish wines may be a source of potentially probiotic yeasts and based on our results we suggest 15_Hans_uvarum, 16_Hans_uvarum and 37_Sacch_cerevisiae as encouraging probiotic yeast candidates with perspectives for further research in aspects including food science, development of probiotic formulations, etc. 

## Figures and Tables

**Table 1:** Code names for tested strains.

Strain Number	Species	Code Name	Vineyard ^1^
01	*Metschnikowia pulcherrima*	01_Metsch_pulcherrima	DB
02	*Starmerella bacillaris*	02_Starm_bacillaris	DB
03	*Saccharomyces cerevisiae*	03_Sacch_cerevisiae	DB
04	*Hanseniaspora uvarum*	04_Hans_uvarum	DB
05	*Hanseniaspora uvarum*	05_Hans_uvarum	DB
06	*Saccharomyces cerevisiae*	06_Sacch_cerevisiae	DB
07	*Hanseniaspora uvarum*	07_Hans_uvarum	DB
08	*Starmerella bacillaris*	08_Starm_bacillaris	DB
09	*Saccharomyces cerevisiae*	09_Sacch_cerevisiae	DB
10	*Saccharomyces cerevisiae*	10_Sacch_cerevisiae	DB
11	*Saccharomyces cerevisiae*	11_Sacch_cerevisiae	DB
12	*Hanseniaspora uvarum*	12_Hans_uvarum	DB
13	*Metschnikowia ziziphicola*	13_Metsch_ziziphicola	MD
14	*Hanseniaspora uvarum*	14_Hans_uvarum	MD
15	*Hanseniaspora uvarum*	15_Hans_uvarum	MD
16	*Hanseniaspora uvarum*	16_Hans_uvarum	MD
17	*Metschnikowia pulcherrima*	17_Metsch_pulcherrima	MD
18	*Metschnikowia pulcherrima*	18_Metsch_pulcherrima	MD
19	*Starmerella bacillaris*	19_Starm_bacillaris	MD
20	*Starmerella bacillaris*	20_Starm_bacillaris	MD
21	*Metschnikowia pulcherrima*	21_Metsch_pulcherrima	MD
22	*Starmerella bacillaris*	22_Starm_bacillaris	WJ
23	*Hanseniaspora uvarum*	23_Hans_uvarum	WJ
24	*Hanseniaspora uvarum*	24_Hans_uvarum	WJ
25	*Saccharomyces cerevisiae*	25_Sacch_cerevisiae	DB
26	*Saccharomyces cerevisiae*	26_Sacch_cerevisiae	DB
27	*Saccharomyces cerevisiae*	27_Sacch_cerevisiae	DB
31	*Starmerella bacillaris*	31_Starm_bacillaris	DB
32	*Pichia kluyveri*	32_Pich_kluyveri	DB
33	*Hanseniaspora uvarum*	33_Hans_uvarum	DB
34	*Metschnikowia pulcherrima*	34_Metsch_pulcherrima	DB
35	*Saccharomyces cerevisiae*	35_Sacch_cerevisiae	DB
36	*Metschnikowia pulcherrima*	36_Metsch_pulcherrima	DB
37	*Saccharomyces cerevisiae*	37_Sacch_cerevisiae	DB
38	*Saccharomyces cerevisiae*	38_Sacch_cerevisiae	DB
39	*Saccharomyces cerevisiae*	39_Sacch_cerevisiae	DB
40	*Saccharomyces cerevisiae*	40_Sacch_cerevisiae	DB
41	*Starmerella bacillaris*	41_Starm_bacillaris	MD
42	*Starmerella bacillaris*	42_Starm_bacillaris	MD
43	*Starmerella bacillaris*	43_Starm_bacillaris	MD
44	*Pichia kluyveri*	44_Pich_kluyveri	MD
45	*Starmerella bacillaris*	45_Starm_bacillaris	MD
46	*Metschnikowia pulcherrima*	46_Metsch_pulcherrima	MD
47	*Starmerella bacillaris*	47_Starm_bacillaris	MD
-	*Saccharomyces cerevisiae* var. *bouardi*	Sacch_boulardi	-

^1^ Vineyards: “Dom Bliskowice” (DB); “Małe Dobre” (MD); and “Winnica Janowiec” (WJ).

**Table 2:** Results of API-ZYM assay for selected yeast strains.

Enzyme	13 ^1^	15 ^1^	16 ^1^	17 ^1^	27 ^1^	32 ^1^	33 ^1^	34 ^1^	35 ^1^	36 ^1^	37 ^1^
Lipase (C14)	-	-	-	-	+	-	-	-	-	-	-
Leucine arylamidase	-	+	-	+	+	+	-	+	+	+	+
Valine arylamidase	-	+	-	+	+	+	-	+	-	+	+
Cystine arylamidase	-	-	-	+	-	+	-	+	-	+	-
Chymotrypsin	-	+	-	+	-	+	-	-	-	+	-
Acid phosphatase	+	+	+	+	+	+	+	+	+	+	+
Naphthol-AS-BI-phosphohydrolase	+	+	+	+	+	+	+	-	+	+	-
β-Galactosidase	+	-	+	-	-	-	+	+	+	-	-
α-Glucosidase	+	+	+	+	+	+	-	+	+	+	+
β-Glucosidase	+	-	+	+	+	+	-	-	-	+	+
N-acetyl-β-glucosaminidase	+	+	-	-	-	-	-	+	+	-	-
α-Mannosidase	-	+	-	+	+	+	-	+	+	+	+

^1^ Strain number (see Table 1).

## Data Availability

Data will be made available on request.

## Supplementary Materials

The following supporting information can be downloaded at: https://www.mdpi.com/article/10.3390/foods12183392/s1, Spreadsheet S1: Figure_5_Tukey_results, Figure_6_Tukey_results.

## References

[1] Sanders M.E., Merenstein D.J., Reid G., Gibson G.R., Rastall R.A. (2019). Probiotics and Prebiotics in Intestinal Health and Disease: From Biology to the Clinic. Nat. Rev. Gastroenterol. Hepatol..

[2] Wang B., Yao M., Lv L., Ling Z., Li L. (2017). The Human Microbiota in Health and Disease. Engineering.

[3] Morelli L., Capurso L. (2012). FAO/WHO Guidelines on Probiotics: 10 Years Later. J. Clin. Gastroenterol..

[4] Staniszewski A., Kordowska-Wiater M. (2021). Probiotic and Potentially Probiotic Yeasts—Characteristics and Food Application. Foods.

[5] Jarocki P., Komoń-Janczara E., Młodzińska A., Sadurski J., Kołodzińska K., Łaczmański Ł., Panek J., Frąc M. (2023). Occurrence and Genetic Diversity of Prophage Sequences Identified in the Genomes of L. Casei Group Bacteria. Sci. Rep..

[6] McFarland L.V. (2015). From Yaks to Yogurt: The History, Development, and Current Use of Probiotics. Clin. Infect. Dis..

[7] Czerucka D., Piche T., Rampal P. (2007). Review Article: Yeast as Probiotics–Saccharomyces Boulardii. Aliment. Pharmacol. Ther..

[8] Zhang F., Aschenbrenner D., Yoo J.Y., Zuo T. (2022). The Gut Mycobiome in Health, Disease, and Clinical Applications in Association with the Gut Bacterial Microbiome Assembly. Lancet Microbe.

[9] Abid R., Waseem H., Ali J., Ghazanfar S., Ali G.M., Elasbali A.M., Alharethi S.H. (2022). Probiotic Yeast Saccharomyces: Back to Nature to Improve Human Health. J. Fungi.

[10] Chen L.S., Ma Y., Maubois J.L., He S.H., Chen L.J., Li H.M. (2010). Screening for the Potential Probiotic Yeast Strains from Raw Milk to Assimilate Cholesterol. Dairy Sci. Technol..

[11] Arévalo-Villena M., Fernandez-Pacheco P., Castillo N., Bevilacqua A., Briones Pérez A. (2018). Probiotic Capability in Yeasts: Set-up of a Screening Method. LWT.

[12] Fernandez-Pacheco Rodríguez P., Arévalo-Villena M., Zaparoli Rosa I., Briones Pérez A. (2018). Selection of Potential Non-Sacharomyces Probiotic Yeasts from Food Origin by a Step-by-Step Approach. Food Res. Int..

[13] Angulo M., Ramos A., Reyes-Becerril M., Guerra K., Monreal-Escalante E., Angulo C. (2023). Probiotic *Debaryomyces hansenii* CBS 8339 Yeast Enhanced Immune Responses in Mice. 3 Biotech.

[14] Homayouni-Rad A., Azizi A., Oroojzadeh P., Pourjafar H. (2020). Kluyveromyces Marxianus as a Probiotic Yeast: A Mini-Review. Curr. Nutr. Food Sci..

[15] Koshchayev I., Lavrinenko K., Medvedeva P. (2022). Probiotic Drug Based on Kluyveromyces Marxianus for Poultry. E3S Web Conf..

[16] Ponomarova O., Gabrielli N., Sévin D.C., Mülleder M., Zirngibl K., Bulyha K., Andrejev S., Kafkia E., Typas A., Sauer U. (2017). Yeast Creates a Niche for Symbiotic Lactic Acid Bacteria through Nitrogen Overflow. Cell Syst..

[17] Sybesma W., Kort R., Lee Y.K. (2015). Locally Sourced Probiotics, the next Opportunity for Developing Countries?. Trends Biotechnol..

[18] Tamang J.P., Lama S. (2022). Probiotic Properties of Yeasts in Traditional Fermented Foods and Beverages. J. Appl. Microbiol..

[19] Kunyeit L., Rao R.P., Anu-Appaiah K.A. (2023). Yeasts Originating from Fermented Foods, Their Potential as Probiotics and Therapeutic Implication for Human Health and Disease. Crit. Rev. Food Sci. Nutr..

[20] de Miranda N.M.Z., de Souza A.C., de Souza Costa Sobrinho P., Dias D.R., Schwan R.F., Ramos C.L. (2023). Novel Yeasts with Potential Probiotic Characteristics Isolated from the Endogenous Ferment of Artisanal Minas Cheese. Brazilian J. Microbiol..

[21] Pytka M., Kordowska-Wiater M., Wajs J., Glibowski P., Sajnaga E. (2022). Usefulness of Potentially Probiotic *L. lactis* Isolates from Polish Fermented Cow Milk for the Production of Cottage Cheese. Appl. Sci..

[22] Cioch-Skoneczny M., Satora P., Skoneczny S., Skotniczny M. (2021). Biodiversity of Yeasts Isolated during Spontaneous Fermentation of Cool Climate Grape Musts. Arch. Microbiol..

[23] García-Ruiz A., González de Llano D., Esteban-Fernández A., Requena T., Bartolomé B., Moreno-Arribas M.V. (2014). Assessment of Probiotic Properties in Lactic Acid Bacteria Isolated from Wine. Food Microbiol..

[24] Marzano M., Fosso B., Manzari C., Grieco F., Intranuovo M., Cozzi G., Mulè G., Scioscia G., Valiente G., Tullo A. (2016). Complexity and Dynamics of the Winemaking Bacterial Communities in Berries, Musts, and Wines from Apulian Grape Cultivars through Time and Space. PLoS ONE.

[25] Kordowska-Wiater M., Pytka M., Stój A., Kubik-Komar A., Wyrostek J., Waśko A. (2022). A Metagenetic Insight into Microbial Diversity of Spontaneously Fermented Polish Red Wines and an Analysis of Selected Physicochemical Properties. Appl. Sci..

[26] Sirén K., Mak S.S.T., Melkonian C., Carøe C., Swiegers J.H., Molenaar D., Fischer U., Thomas P., Gilbert M. (2019). Taxonomic and Functional Characterization of the Microbial Community During Spontaneous in Vitro Fermentation of Riesling Must. Front. Microbiol..

[27] Vergara Alvarez S.C., Leiva Alaniz M.J., Mestre Furlani M.V., Vazquez F., Mancha Agresti P., Cristina Nally M., Paola Maturano Y. (2023). Bioprospecting of the Probiotic Potential of Yeasts Isolated from a Wine Environment. Fungal Genet. Biol..

[28] Vilela A., Cosme F., Inês A. (2020). Wine and Non-Dairy Fermented Beverages: A Novel Source of Pro-and Prebiotics. Fermentation.

[29] Rayavarapu B., Tallapragada P. (2019). Evaluation of Potential Probiotic Characters of *Lactobacillus fermentum*. Sci. Study Res. Chem. Chem. Eng. Biotechnol. Food Ind..

[30] Agarbati A., Canonico L., Marini E., Zannini E., Ciani M., Comitini F. (2020). Potential Probiotic Yeasts Sourced from Natural Environmental and Spontaneous Processed Foods. Foods.

[31] Przybek P. Winnice w Polsce Mapa 581 Polskich Winnic Winogrodnicy.PL. http://winogrodnicy.pl/.

[32] Drozdz I., Makarewicz M., Sroka P., Satora P., Jankowski P. (2015). Comparison of the Yeast Microbiota of Different Varieties of Cool-Climate Grapes by PCR-RAPD. Potravin. Slovak J. Food Sci..

[33] White T.J., Bruns T.D., Lee S.B., Taylor J.W. (1990). Amplification and Direct Sequencing of Fungal Ribosomal RNA Genes for Phylogenetics. PCR-Protocols and Applications—A Laboratory Manual.

[34] Altschul S.F., Boguski M.S., Gish W., Wootton J.C. (1994). Issues in Searching Molecular Sequence Databases. Nat. Genet..

[35] Sanders E.R. (2012). Aseptic Laboratory Techniques: Plating Methods. J. Vis. Exp..

[36] Buck J.D., Cleverdon R.C. (1960). The Spread Plate As A Method For The Enumeration Of Marine Bacteria. Limnol. Oceanogr..

[37] Amorim J.C., Piccoli R.H., Duarte W.F. (2018). Probiotic Potential of Yeasts Isolated from Pineapple and Their Use in the Elaboration of Potentially Functional Fermented Beverages. Food Res. Int..

[38] Perricone M., Bevilacqua A., Corbo M.R., Sinigaglia M. (2014). Technological Characterization and Probiotic Traits of Yeasts Isolated from Altamura Sourdough to Select Promising Microorganisms as Functional Starter Cultures for Cereal-Based Products. Food Microbiol..

[39] Syal P., Vohra A. (2013). Probiotic Potential of Yeasts Isolated From Traditional Indian Fermented Foods. Int. J. Microbiol. Res..

[40] Gil-Rodríguez A.M., Carrascosa A.V., Requena T. (2015). Yeasts in Foods and Beverages: In Vitro Characterisation of Probiotic Traits. LWT-Food Sci. Technol..

[41] Magaldi S., Mata-Essayag S., Hartung de Capriles C., Perez C., Colella M., Olaizola C., Ontiveros Y., Ellis M., Ain A. (2004). Well Diffusion for Antifungal Susceptibility Testing. Int. J. Infect. Dis..

[42] Balouiri M., Sadiki M., Ibnsouda S.K. (2016). Methods for in Vitro Evaluating Antimicrobial Activity: A Review. J. Pharm. Anal..

[43] Aslankoohi E., Herrera-Malaver B., Rezaei M.N., Steensels J., Courtin C.M., Verstrepen K.J. (2016). Non-Conventional Yeast Strains Increase the Aroma Complexity of Bread. PLoS ONE.

[44] Jung M.Y., Lee C., Seo M.J., Roh S.W., Lee S.H. (2020). Characterization of a Potential Probiotic Bacterium *Lactococcus raffinolactis* WiKim0068 Isolated from Fermented Vegetable Using Genomic and in Vitro Analyses. BMC Microbiol..

[45] Bautista-Gallego J., Arroyo-López F.N., Rantsiou K., Jiménez-Díaz R., Garrido-Fernández A., Cocolin L. (2013). Screening of Lactic Acid Bacteria Isolated from Fermented Table Olives with Probiotic Potential. Food Res. Int..

[46] Pan W.H., Li P.L., Liu Z. (2006). The Correlation between Surface Hydrophobicity and Adherence of Bifidobacterium Strains from Centenarians’ Faeces. Anaerobe.

[47] Pizzolitto R.P., Armando M.R., Salvano M.A., Dalcero A.M., Rosa C.A. (2013). Evaluation of *Saccharomyces cerevisiae* as an Antiaflatoxicogenic Agent in Broiler Feedstuffs. Poult. Sci..

[48] Nadai C., Giacomini A., Corich V. (2021). The Addition of Wine Yeast *Starmerella bacillaris* to Grape Skin Surface Influences Must Fermentation and Glycerol Production. OENO One.

[49] Englezos V., Giacosa S., Rantsiou K., Rolle L., Cocolin L. (2017). *Starmerella bacillaris* in Winemaking: Opportunities and Risks. Curr. Opin. Food Sci..

[50] Masneuf-Pomarede I., Juquin E., Miot-Sertier C., Renault P., Laizet Y., Salin F., Alexandre H., Capozzi V., Cocolin L., Colonna-Ceccaldi B. (2015). The Yeast *Starmerella bacillaris* (Synonym *Candida zemplinina*) Shows High Genetic Diversity in Winemaking Environments. FEMS Yeast Res..

[51] Drumonde-Neves J., Franco-Duarte R., Lima T., Schuller D., Pais C. (2016). Yeast Biodiversity in Vineyard Environments Is Increased by Human Intervention. PLoS ONE.

[52] Pretorius I.S., Van der Westhuizen T.J., Augustyn O.P.H. (1999). Yeast Biodiversity in Vineyards and Wineries and Its Importance to the South African Wine Industry: A Review. S. Afr. J. Enol. Vitic..

[53] Capozzi V., Garofalo C., Chiriatti M.A., Grieco F., Spano G. (2015). Microbial Terroir and Food Innovation: The Case of Yeast Biodiversity in Wine. Microbiol. Res..

[54] Shen Y., Bai X., Zhang Y., Gao Q., Bu X., Xu Y., Guo N. (2022). Evaluation of the Potential Probiotic Yeast Characteristics with Anti-MRSA Abilities. Probiotics Antimicrob. Proteins.

[55] Turk M., Abramović Z., Plemenitaš A., Gunde-Cimerman N. (2007). Salt Stress and Plasma-Membrane Fluidity in Selected Extremophilic Yeasts and Yeast-like Fungi. FEMS Yeast Res..

[56] Menezes A.G.T., Ramos C.L., Cenzi G., Melo D.S., Dias D.R., Schwan R.F. (2020). Probiotic Potential, Antioxidant Activity, and Phytase Production of Indigenous Yeasts Isolated from Indigenous Fermented Foods. Probiotics Antimicrob. Proteins.

[57] Sipiczki M. (2020). *Metschnikowia pulcherrima* and Related Pulcherrimin-Producing Yeasts: Fuzzy Species Boundaries and Complex Antimicrobial Antagonism. Microorganisms.

[58] Tenea G.N., Anrango Cajas B., Carlosama Sanchez B. (2023). Inhibitory-like Substances Produced by Yeasts Isolated from Andean Blueberries: Prospective Food Antimicrobials. Foods.

[59] Corbu V.M., Csutak O. (2023). Molecular and Physiological Diversity of Indigenous Yeasts Isolated from Spontaneously Fermented Wine Wort from Ilfov County, Romania. Microorganisms.

[60] Fernández-Pacheco P., Ramos Monge I.M., Fernández-González M., Poveda Colado J.M., Arévalo-Villena M. (2021). Safety Evaluation of Yeasts With Probiotic Potential. Front. Nutr..

[61] Matukas M., Starkute V., Zokaityte E., Zokaityte G., Klupsaite D., Mockus E., Rocha J.M., Ruibys R., Bartkiene E. (2022). Effect of Different Yeast Strains on Biogenic Amines, Volatile Compounds and Sensory Profile of Beer. Foods.

[62] Stój A., Płotka-Wasylka J., Simeonov V., Kapłan M. (2022). The Content of Biogenic Amines in Rondo and Zweigelt Wines and Correlations between Selected Wine Parameters. Food Chem..

[63] Delgado-Ospina J., Acquaticci L., Molina-Hernandez J.B., Rantsiou K., Martuscelli M., Kamgang-Nzekoue A.F., Vittori S., Paparella A., Chaves-López C. (2020). Exploring the Capability of Yeasts Isolated from Colombian Fermented Cocoa Beans to Form and Degrade Biogenic Amines in a Lab-Scale Model System for Cocoa Fermentation. Microorganisms.

[64] Caruso M., Fiore C., Contursi M., Salzano G., Paparella A., Romano P. (2002). Formation of Biogenic Amines as Criteria for the Selection of Wine Yeasts. World J. Microbiol. Biotechnol..

[65] Landete J.M., Ferrer S., Pardo I. (2007). Biogenic Amine Production by Lactic Acid Bacteria, Acetic Bacteria and Yeast Isolated from Wine. Food Control.

[66] Tanner A.C.R., Strzempko M.N., Belsky C.A., McKinley G.A. (1985). API ZYM and API An-Ident Reactions of Fastidious Oral Gram-Negative Species. J. Clin. Microbiol..

[67] Monteagudo-Mera A., Caro I., Rodriguez-Aparicio L.B., Rua J., Ferrero M.A., Garcia-Armesto M.R. (2011). Characterization of Certain Bacterial Strains for Potential Use as Starter or Probiotic Cultures in Dairy Products. J. Food Prot..

[68] Valderrama B., Ruiz J.J., Gutiérrez M.S., Alveal K., Caruffo M., Oliva M., Flores H., Silva A., Toro M., Reyes-Jara A. (2021). Cultivable Yeast Microbiota from the Marine Fish Species *Genypterus chilensis* and *Seriolella violacea*. J. Fungi.

[69] Lee N.-K., Hong J.-Y., Yi S.-H., Hong S.-P., Lee J.-E., Paik H.-D. (2019). Bioactive Compounds of Probiotic *Saccharomyces cerevisiae* Strains Isolated from Cucumber *Jangajji*. J. Funct. Foods.

[70] Patil R.S., Ghormade V., Deshpande M.V. (2000). Chitinolytic Enzymes: An Exploration. Enzyme Microb. Technol..

